# Knowledge, attitudes, and practices of community pharmacists toward the management of acne vulgaris in Saudi Arabia

**DOI:** 10.3389/fmed.2023.1133765

**Published:** 2023-06-27

**Authors:** Ziyadh Alrabiah, Syed Wajid, Salmeen D. Babelghaith, Mohamed N. Al Arifi

**Affiliations:** Department of Clinical Pharmacy, College of Pharmacy, King Saud University, Riyadh, Saudi Arabia

**Keywords:** acne vulgaris, knowledge, attitudes, community pharmacists, Saudi Arabia

## Abstract

**Background and objectives:**

In Saudi Arabia, Acne vulgaris is a very predominant ailment and Pharmacists currently have standardized protocols at their disposal for the treatment of acne. Pharmacists often prescribe medications for minor cases of acne. Therefore, this study aimed to explore community pharmacists (CPs)’ knowledge, attitudes, and practice toward acne management in Riyadh, Saudi Arabia.

**Methods:**

An online cross-sectional study was conducted among CPs working in Riyadh, Saudi Arabia from January and April 2021, using a self-administered, questionnaire, was divided into four sections that collected information from the CP’s Knowledge, attitudes, and practices and the management of acne vulgaris. The data were analyzed using the Statistical Package for the Social Sciences version 26 (SPSS).

**Result:**

A total of 313 CPs were enrolled in the study who successfully returned the questionnaire. The mean score of knowledge on etiology, pathophysiology, and therapy for AV was 5.3 ± (SD = 1.5). However, the majority of CPs had a moderate level of knowledge (80.8%), followed by mild to moderate (12.8%). This study showed that CPs had an insufficient level of knowledge about ace management, about 6% of CPs have a high level of knowledge. Inadequate knowledge was revealed in their management practice, only 0.3% of CPs treated patients with acne on their own without a referral. In addition, CPs showed a positive attitude toward acne management.

**Conclusion:**

There is a need to improve CPs’ understanding of acne care and to contribute to participating in organized training sessions on the management of AV.

## Introduction

Acne vulgaris (AV) is a major skin disorder, 85% of adolescents between the ages of 15 and 17 have acne vulgaris; though, acne can affect anyone (1, 2). A chronic inflammatory pilosebaceous condition known as AV causes lesions on the skin in regions with a high concentration of big sebaceous glands, such as the face, chest, shoulders, and upper back (2). Age of the patient, lesion shape (comedonal, inflammatory, mixed, nodulocystic), distribution (facial, trunk, or both), and severity are used to categorize acne vulgaris (extent, presence or absence of scarring, post-inflammatory erythema, or hyperpigmentation) (2).

Although the majority of acne cases do not require a specific medical evaluation, a medical workup may occasionally be necessary (2–5). An estimated 9.4% of the global population is impacted by acne and the majority of them were being female (4–6). A more recent study in Saudi Arabia reported a prevalence of 78.5% of acne among university students (6). On the other hand, the American Academy of Dermatology revealed that acne impacted approximately 50 million Americans annually. Approximately 85% of acne sufferers were between the ages of 12 and 24 and making it the eighth most common disease on the planet (7). Additionally, patients who encounter side effects require continuous treatment, which increases the expense (3, 8–10). First-line treatments include topical therapy such as retinoid (such as tretinoin, and adapalene), benzoyl peroxide, azelaic acid, and/or combinations of topical medications (11). In a randomized trial including 207 participants, treatment with tretinoin 0.025% gel reduced the number of acne lesions at 12 weeks by 63% compared to baseline (11). Oral hormonal therapy such as combined oral conceptive drugs or spironolactone, oral antibiotics like doxycycline or minocycline, or Isotretinoin is most effective for more severe diseases (11). The studies on pharmacists’ knowledge and attitude toward acne management are more difficult to obtain since there are only a few studies on the topic throughout Saudi Arabia. Most studies have discussed the knowledge and practice of medical doctors regarding acne management. Previous studies in Saudi Arabia among healthcare providers’ reported low knowledge of acne management (1, 12, 13). Among those studies, a study by Khalid et al. (12) examined the knowledge, attitudes, and practices of doctors working in primary health care centers about acne vulgaris. It was a cross-sectional study carried out in 30 PHCs in Riyadh in 2000 and involved 144 physicians (13). Inappropriate behavior and a lack of understanding among the health care professionals were the outcomes that were associated with lower knowledge of acne. On the other hand, another cross-sectional by Al-Shobaili et al., in 2012 in the Qassim region conducted among physicians working at Primary Health Care facilities and reported that there was a lack of knowledge and practice of acne (1). Similarly, another study by Alotaibi et al., carried out to assess the knowledge and practice of primary healthcare physicians for the management of Acne Vulgaris in the Sudair area in Saudi Arabia, found that 49% of physicians had good knowledge, 44% had moderate knowledge and 7% had poor knowledge (12).

Community pharmacists are frontline healthcare workers and are regarded as experts in patient education and counseling (14–20). This includes gathering a patient’s medical and medication history, observing the quantity and kind of lesions, choosing the best therapeutic plan, counseling the patient, and, if necessary, referring the patient to a doctor. Patients must be informed about the treatment’s objectives, reasonable expectations, length of therapy, appropriate product use, and the significance of following the regimen to reap the greatest benefits (14–17). Community pharmacists are in a prime position to dispense the best course of action for people with mild-to-moderate illnesses and refer people with severe instances for additional evaluation (14–17). As far as we are aware, no such study or survey has been carried out in Saudi Arabia to explore the knowledge, practice, and attitude of CPs on acne management. This study was undertaken among pharmacists working at CPs in central Saudi Arabia to examine their knowledge, attitude, and practice for acne management because pharmacist knowledge is crucial to the proper treatment of AV and its psychological effects.

## Methods

### Study design

This was a cross-sectional study conducted between January and April 2021, among community pharmacies in Riyadh city, Saudi Arabia. Written informed consent was taken from the participants before their inclusion in the survey. The sampling was random.

### Sample size

The sample of registered CPs in Riyadh was estimated to be 2000 CPs (21). Similar to previous studies (14, 22–28) the number of samples was calculated using the Raosoft online calculator (5). To obtain a 95% confidence interval level and a 5% accepted margin of error when taking into account a 50% answer distribution, an estimated 323 participants were needed. For this research, we chose pharmacies in several Riyadh regions. Covering the suburbs in the North, South, West, East, and Center.

### Questionnaire

A paper-based self-administered questionnaire was used to collect data. The questionnaire utilized in this study was created after a thorough evaluation of the literature on health (2, 3, 29). The questionnaire was divided into four components; the first section asked about sociodemographics. The second section included 10 items that were used to evaluate the pharmacists’ knowledge in treating acne. Each correct response received one point, whereas a wrong response received zero points. The levels were classified as low level (0–3), moderate level (4–7), and high level (8–10). The third section included five questions to determine the pharmacists’ practices in counseling and treating acne patients. The fourth section included five questions to examine the attitudes of the pharmacists treating acne. A panel of four clinical pharmacists with expertise in pharmaceutical research examined the questionnaire’s content for face validity, and it was piloted with 10 CPs to determine how long it would take to administer and to check for clarity and dependability. The surveys utilized in the final statistical analysis did not take the pilot study into account.

### Data analysis

The data was analyzed using Statistical Package for Social Sciences version 26.0 (SPSS Inc., Chicago, IL, United States). Chi-squared and Fisher’s test were used to find the association between categorical variables. To find out the predictors effecting on the knowledge score of Acne among CPs we used simple linear multiple regression analysis at *p* < 0.05 significant level.

## Results

A total of 313 CPs were enrolled in the study and successfully returned the questionnaire. Most CPs were between 25 and 35 years of age. Most of them (34.8%) had less than 5 years of practice in a community pharmacy setting. The demographic information gathered is summarized in Table 1.

**Table 1:** Demographic data (*N* = 313).

Items	Frequency (*n*)	Percentage (%)
Age		
25–35	204	65.2%
36–45	80	25.6%
46–55	27	8.6%
56–65	2	0.6%
Pharmacy type		
Chain community pharmacy	278	91.7%
Independent community pharmacy	26	8.3%
Work experiences		
<6 months	35	11.2%
>6 months	42	13.4%
>1 year	51	16.3%
>2 years	185	59.1%
Years in practice		
<1 year	36	11.5%
1 < 5 years	76	24.3%
5 < 10 years	109	34.8%
10 to <20 years	78	24.9%
20 years or more	14	4.5%
Graduation country*		
Saudi Arabia	66	21.1%
Egypt	189	60.4%
Sudan	21	6.7%
Yemen	8	2.6%
Other	14	4.5%

*Missing data.

This study found that the mean score of knowledge on etiology, pathophysiology, and therapy for AV was 5.3 ± 1.5. However, the majority of participants had a moderate level of knowledge (80.8%), followed by mild–moderate (12.8%). The total knowledge levels were low 12.8% (*n* = 40), moderate 80.8% (*n* = 253), and high 6.4% (*n* = 20).

Table 2 shows the CP’s responses to questions on the physiological issues and causes of acne. The most of causes of acne were hormones (82.7%), followed by bacteria (81.5%), stress (62.9%), and cosmetics (35.4%). The majority of the CPs stated that topical antibiotics are most frequently used to treat acne in general (87.5%), followed by oral Antibiotics (78.3%), and topical retinoids (73.8%). On the other hand, 93% of the pharmacists agreed that topical antifungal drugs should not be used to treat acne (Table 2). A Fishers Exact test was run at a significance level of 0.05 to establish the relationship between the socio-demographic characteristics of the participants and knowledge levels. The results showed no difference, except the type of pharmacy where the CPs work (*p* = 0.043) was found as presented in Table 3.

**Table 2:** Knowledge of community pharmacist about acne.

Variable	Frequency (%)
Causes of acne vulgaris*	
Cosmetic	112 (35.4%)
Bacteria	255 (81.5%)
Hormones	259 (82.7%)
Stress	197 (62.9%)
The drug commonly used in the management of acne*	
Oral antibiotics	245 (78.3%)
Topical antibiotics	274 (87.5%)
Topical steroids	44 (14.1%)
Topical retinoid	231 (73.8%)
Topical antihistamine	20 (6.4%)
Topical antifungal	22 (7.0%)

* Multiple-choice questions.

**Table 3:** Correlation of knowledge level with socio-demographic characteristics of the participants.

Variables	Knowledge level			*p* value
	Low *N* (%) 40 (12.8)	Moderate *N* (%) 253 (80.8)	High *N* (%) 20 (6.4)	
Age				0.103*
25–35	21 (10.3)	169 (82.8)	14 (6.9)	
36–45	10 (12.5)	66 (82.5)	4 (5.0)	
46–55	8 (29.6)	17 (63.0)	2 (7.4)	
56–65	1 (50.0)	1 (50.0)	0	
Pharmacy type				0.043
Chain community pharmacy	33 (11.5)	234 (81.5)	20 (7.0)	
Independent community pharmacy	7 (26.9)	19 (73.1)	–	
Work experiences				0.238*
<6 months	69 (17.1)	27 (77.1)	2 (5.7)	
>6 months	7 (16.7)	32 (76.2)	39 (7.1)	
>1 year	11 (21.6)	38 (74.5)	2 (3.9)	
>2 years	16 (8.6)	156 (84.3)	13 (7.0)	
Years in practice				0.72*
<1 year	5 (13.9)	30 (83.3)	1 (2.8)	
1 < 5 years	11 (14.5)	75 (75.0)	8 (10.5)	
5 < 10 years	12 (11.0)	92 (84.4)	5 (4.6)	
10 to <20 years	9 (11.5)	64 (82.1)	5 (6.4)	
20 years or more	3 (21.4)	10 (71.4)	1 (7.1)	
Graduation country*				0.141*
Saudi Arabia	6 (9.1)	55 (83.3)	5 (7.6)	
Egypt	22 (11.6)	155 (82.0)	12 (6.3)	
Sudan	7 (33.3)	14 (66.7)	0	
Yemen	2 (25.00)	5 (62.5)	1 (12.5)	
Other	3 (21.4)	10 (71.4)	1 (7.1)	

*Fisher exact test.

The results of the simple linear multiple regression analysis revealed that, type of pharmacy where CPs were worked, years of practice and graduation country were not significant predictor of knowledge score (*p* = 0.05), except age of the CPs, which was significant predictor of the knowledge score (*p* = 0.006) of the acne as shown in the Table 4.

**Table 4:** Predictors of acne knowledge score among pharmacists.

Model	Unstandardized coefficients		Standardized coefficients	*t*	Sig.
	*B*	Std. Error	Beta		
(Constant)	5.735	0.508		11.283	<0.001
Age	−0.447	0.163	−0.191	−2.746	0.006
Pharmacy type	−0.566	0.328	−0.099	−1.722	0.086
Work	0.159	0.105	0.110	1.511	0.132
Practice	0.030	0.123	0.020	0.246	0.806
Graduation country	0.086	0.077	0.069	1.123	0.262

Assessing the CPs’ practice toward the management of acne is presented in Table 5. More than one-half of the participants believed that the course of treatment should go longer than 1 month. About 95% of the pharmacists preferred to refer patients with severe acne to a dermatologist, and about 71% of participants agreed to follow up with their patients. In terms of patient counseling for acne, 65% of pharmacists recommended diet changes, 60.1% advised patients to refrain from picking at pimples, 53% advised patients to avoid stress, and 65.8% of CPs instructed their patients to follow treatment regimens (Table 5).

**Table 5:** Community pharmacy’ practice of acne management.

Variables	Frequency (%)
The average duration of treatment for acne*	
1 week	14 (4.5)
2–4 weeks	119 (38.0)
More than 1 month	177 (56.6)
Follow-up acne vulgaris patients*	
Yes	223 (71.2)
No	87 (27.8)
Refer patients with acne to a dermatologist*	
Yes	296 (94.6)
No	10 (3.2)
Treatment patients with severe acne*	
Refer to a dermatologist without any intervention	287 (91.7)
Provide advice only	20 (6.4)
Dispense medication on your own	1 (0.3)
Advice that you provide to the patient with acne vulgaris	
Adhere to the treatment regimen	206 (65.8)
Avoid manipulating pimples	188 (60.1)
Avoid stress	166 (53.0)
Diet modification	204 (65.2)

*Missing data.

### Attitudes toward management of patients with acne

When asked about their attitudes toward managing patients with acne, our participants indicated that they had a positive attitude. About 93% of CPs asked about patients’ medical and prescription histories and only 58.1% of CPs asked patients about their family’s history of acne. The majority of CPs (95.2%) ask patients if they are pregnant or expecting to become pregnant and 91.7% of them inquire about the female patients’ hormonal regularity. In addition, this study found that (Table 6).

**Table 6:** Attitude of pharmacist toward treatment of acne vulgaris.

Variable	Frequency (%)
Inquiring about medication history	
Yes	290 (92.7)
No	23 (7.3)
Inquiring about family history of acne	
Yes	182 (58.1)*
No	131 (41.9)
Inquiring about hormonal regularity	
Yes	287 (91.7)*
No	25 (8.0)
Enquiring about pregnancy	
Yes	298 (95.2)
No	15 (4.8)

*Significant difference at *p* < 0.05 (pharmacy type and graduation country).

## Discussion

Acne vulgaris significantly worsens a patient’s quality of life by impairing their psychosocial development and sense of self-worth. There are numerous over-the-counter and prescription acne therapies available, making it difficult for patients and healthcare professionals to select the most efficient course of treatment (29, 30). Due to its evident appearance, acne is very easy to diagnose, although there is a variety of over-the-counter (OTC) therapies available. Community pharmacies are the perfect place for acne patients to get guidance and treatment because of their accessibility and convenience. However, OTC therapy marketing and the social effects of acne, such as shame and embarrassment, may lead to patient requests or perceived preferences that may influence pharmacy staff recommendations while being inappropriate. Several studies in various contexts have found that acne sufferers have poor awareness of the origins of the problem, and there is even some evidence that local pharmacists have insufficient knowledge of the issue (3, 31). However, to guarantee that the illness is adequately controlled, patients must be educated on self-care and how to apply topical therapies. Many patients are suffering from minor to moderate illnesses that can be easily treated in pharmacies.

In this study, the majority of CPs (80.8%) showed moderate knowledge of acne management. Our results are better than previous studies published among CPs in other countries (2, 3) and primary healthcare providers in Saudi Arabia (1, 12). For instance, a study conducted among CPs in Palestine showed 69.3% had moderate knowledge, and 7.7% had high knowledge (2). Another study from Zambia reported health care providers involving CPs correctly answered questions about the treatment of acne. (3). In Saudi Arabia, a study conducted on primary care medical doctors revealed that 38.7% of doctors had a low level of knowledge, while only 11.3% had a high level of knowledge (1). A more recent study carried out in the center of, Saudi Arabia among primary healthcare physicians found that 49% of them had high while 44% had moderate, and 7% of the physicians were found to have poor knowledge of acne (12).

In the present study, hormone imbalances are the main cause of AV reported by the majority of CPs (82.7%), followed by bacteria (81.5%) and stress (62.9%). This is completely in line with the theory that hormonal variables are the first to cause acne to appear (32, 33). Numerous investigations have shown that *P. acnes* is the cause of acne (32, 34). However, it is also well known that the pathogenesis of acne vulgaris is multifaceted and may involve immunological disorders, genetic changes, stress, and cosmetics the full pathogenesis of acne is, however, not fully understood (35–37).

The goals of acne treatment are to reduce the severity and recurrences of skin lesions as well as to improve the look. The approach is determined by the degree of acne, the patient’s age, treatment preferences, and adherence to and reaction to the prior medication (30). The standard of care for mild to moderate acne is a topical medication (38). The cornerstones of topical acne therapy include retinoids and antimicrobials like benzoyl peroxide and antibiotics. Such therapies can stop the development of new lesions and are active where they are applied (39). In the present study, topical antibiotics and topical retinoids were dispensed by the majority of CPs. These recommendations are in line with the international guidelines which consider topical retinoids should be used as the initial treatment for mild-to-moderate inflammatory acne, either alone or in combination, and that it is also the best drug for maintenance therapy (40).

For practice, more than half of CPs agree that therapy should extend longer than a month. The international guidelines stated that therapy should initiate with first-line medications for 12 weeks (NICE). Even though therapy for acne can effectively alleviate its social, emotional, and psychological side effects, patient adherence to treatment is low (33). Healthcare providers such as pharmacists must acquire the ability to counsel both adults and adolescents to increase adherence. This study revealed that most CPs provided counseling on adherence to medication, diet modification, and stress. This study found that about 95% of CPs stated that they might refer acne patients to a doctor/dermatologist; this high referral rate in our study could be attributed to a lack of emphasis on ongoing care and poor counseling abilities. Only one community pharmacist can treat acne alone in his pharmacy, which reflects a lack of knowledge, counseling abilities, and effective communication. However, a study conducted in Palestine reported that about 25% of CPs can manage patients with acne in their community pharmacy setting (2).

Pharmacists are essential in the patient evaluation process. This includes gathering a patient’s medical and medication history, observing the quantity and kind of lesions, choosing the best therapeutic plan, counseling the patient, and, if necessary, referring the patient to a doctor. Patients must be informed about the treatment’s objectives, reasonable expectations, length of therapy, appropriate product use, and the significance of following the regimen to reap the greatest benefits (41). These protocols help distinguish between individuals who would benefit from treatment in the pharmacy and those who should be sent to other healthcare professionals. There are currently standardized methods available for pharmacists to treat acne (43). Therefore, CPs in Saudi Arabia should be able to communicate well and should advise acne patients on the many treatments available, how to utilize the treatment, how crucial it is to keep to the regimen, and any potential side effects like dryness and irritation.

Though, CPs in the present study had positive attitudes and behaviors about acne treatment. However, several research has shown that patients and providers’ attitudes toward acne and other skin diseases are negatively influenced by socio-psychological factors (22). Around 95% of the CPs inquire the patients about pregnancy, 91.7% inquire the woman patients for hormonal regularity, and 92.7% inquire the patients for their medical and drug history. Pharmacists identified several challenging factors, including poor pharmacist-patient communication due to space constraints in the pharmacy and gender embarrassment, a lack of experience in the management of acne, and difficulty accessing the most recent treatment recommendations for acne (2). All these factors should be addressed and avoided for better prescribing and patient counseling this highlights the need for national guidelines for the management of acne as well as increased continued educational programs for CPs through various seminars and presentations. Furthermore, organizations must provide social and financial support to enlarge their knowledge in the management of various minor ailments. This study has certain limitations. First, it has a small sample size, despite all efforts to include as many Cps as possible. Second, this study comprised CPs from a single region in Saudi Arabia, limiting the generalizability of the results. Despite these limitations, our study has several merits. To begin, this study considers the significance of acne suffers expertise in providing better treatment and QOL for acne sufferers. There is also a scarcity of such research in the field, notably among CPs in this concept in Saudi Arabia.

## Conclusion

The current study demonstrated that CPs have a positive attitude and good practices for treating acne, although they still need to improve their knowledge. Only 6.8% of CPs have high knowledge, whereas the majority of CPs had a moderate level of knowledge about acne. In addition, very few CPs can treat acne in their pharmacy setting. As a result, it is advised to identify the elements that limit CPs’ ability to provide effective acne management. Furthermore, a communication gap between the patient and the CPs caused in poor adherence to tropical treatment. Therefore, is important to improved patient and pharmacist communication aids in comprehending the patient’s issue, resulting in a better treatment outcome. Future research aimed at developing guidelines to improve the communication of dose regimen instructions by community pharmacists to acne patients may aid in the proper management of acne by community pharmacists. There is a need to encourage pharmacists to engage in structured and concentrated acne management professional training programs.

## Data availability statement

The original contributions presented in the study are included in the article/supplementary material, further inquiries can be directed to the corresponding author.

## Ethics statement

Ethical review and approval was not required for the study on human participants in accordance with the local legislation and institutional requirements. The patients/participants provided their written informed consent to participate in this study.

## Author contributions

All authors listed have made a substantial, direct, and intellectual contribution to the work and approved it for publication.

## Funding

The authors of this study extend their appreciation to Researchers Supporting Project (Project number RSP-2023/81), King Saud University, Saudi Arabia.

## Conflict of interest

The authors declare that the research was conducted in the absence of any commercial or financial relationships that could be construed as a potential conflict of interest.

## Publisher’s note

All claims expressed in this article are solely those of the authors and do not necessarily represent those of their affiliated organizations, or those of the publisher, the editors and the reviewers. Any product that may be evaluated in this article, or claim that may be made by its manufacturer, is not guaranteed or endorsed by the publisher.
